# Supplementary figures and images for: Conformational regulation and target-myristoyl switch of calcineurin B homologous protein 3
Source: eLife. 2023 Jul 12;12:e83868. doi: 10.7554/eLife.83868 (PMC10368425; doi:10.7554/eLife.83868)

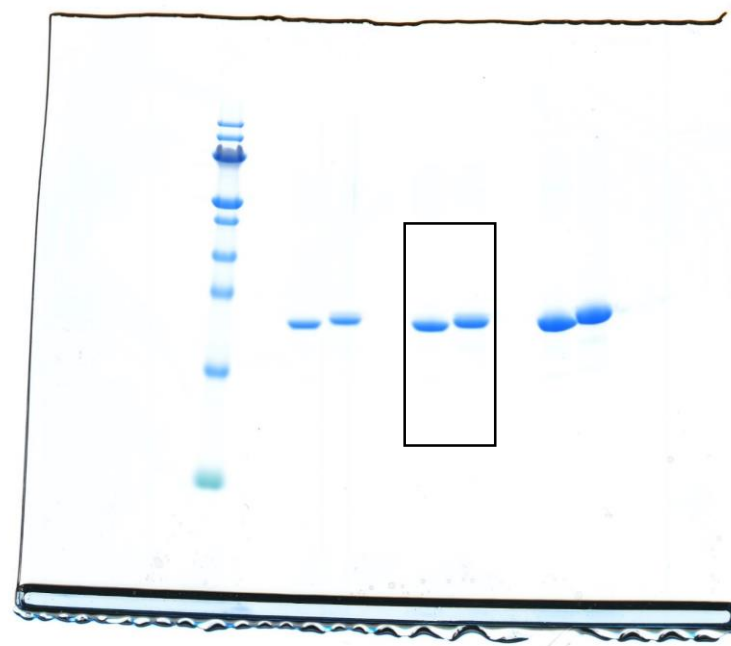

Fig.1A  
mirrored

Supplement: Figure 1—source data 1. [file elife-83868-fig1-data1.zip › Figure1A_labeled.pdf]

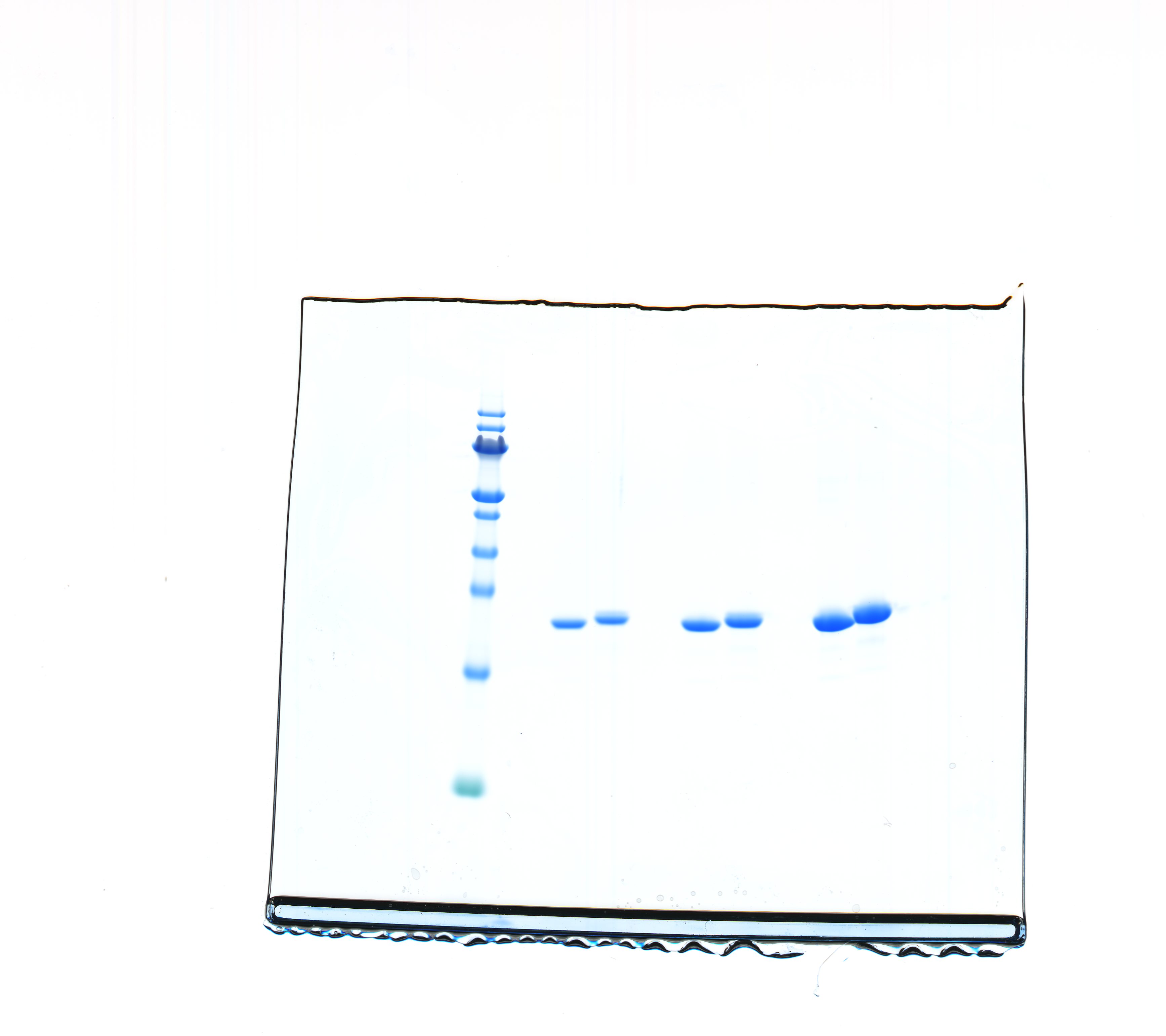

Supplement: Figure 1—source data 1. [file elife-83868-fig1-data1.zip › Figure1A_uncropped.jpg]

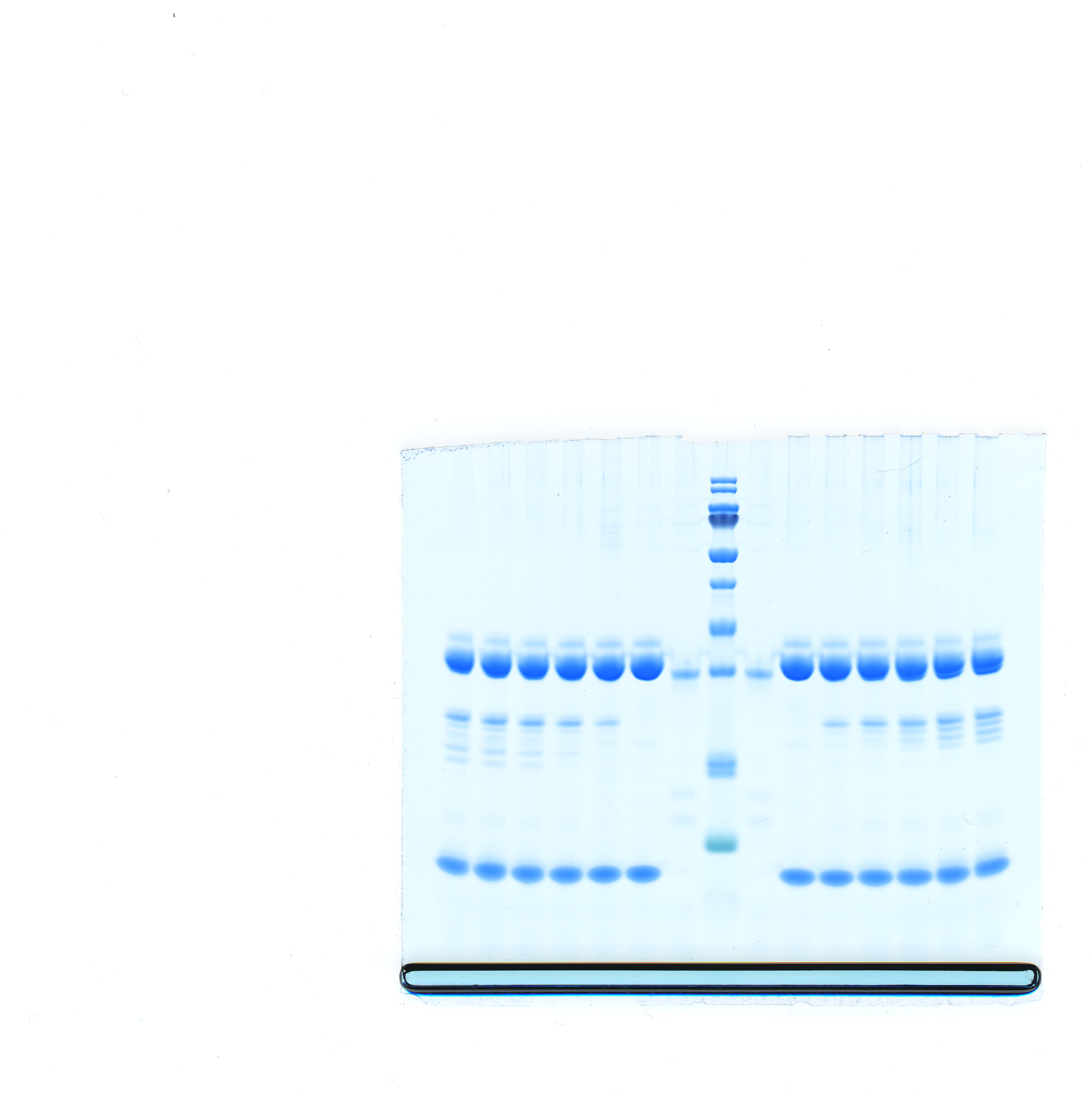

Supplement: Figure 4—source data 1. [file elife-83868-fig4-data1.zip › Fig.4A_bottom_uncropped.jpg]

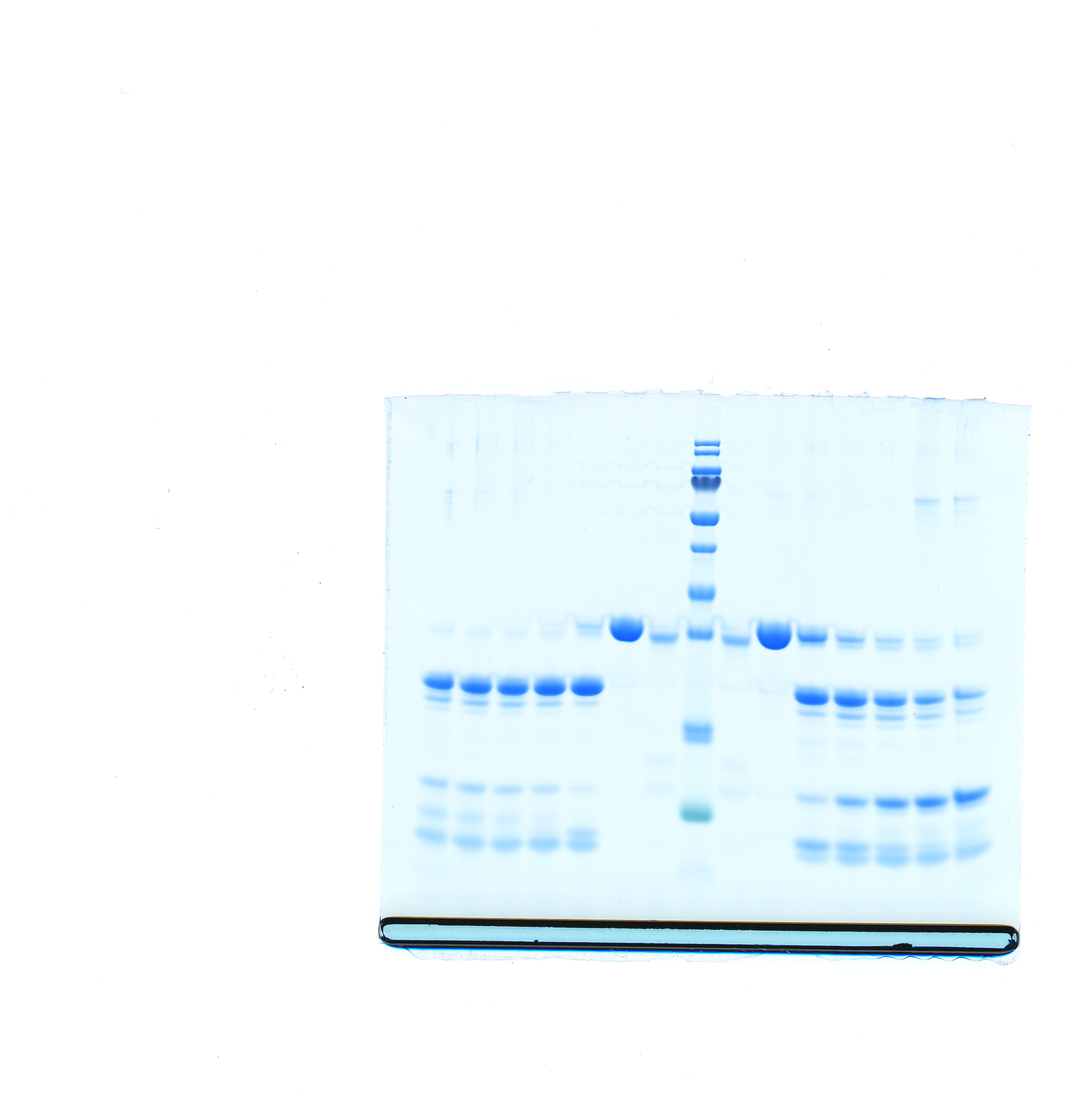

Supplement: Figure 4—source data 1. [file elife-83868-fig4-data1.zip › Fig.4A_top_uncropped.jpg]

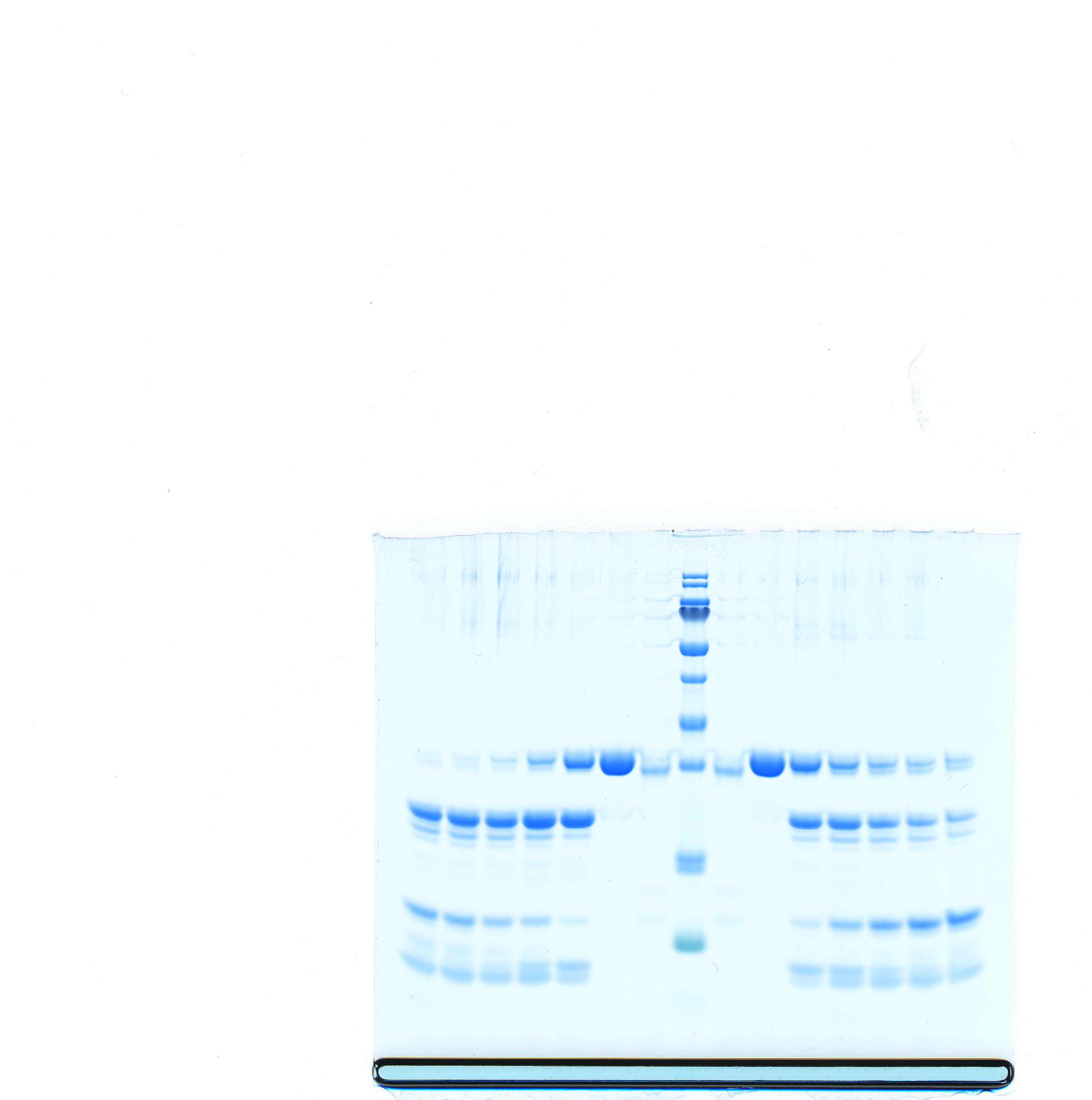

Supplement: Figure 4—figure supplement 1—source data 1. [file elife-83868-fig4-figsupp1-data1.zip › Figure 4-figure supplement 1-CHP3-Ca&Mg-uncropped.jpg]

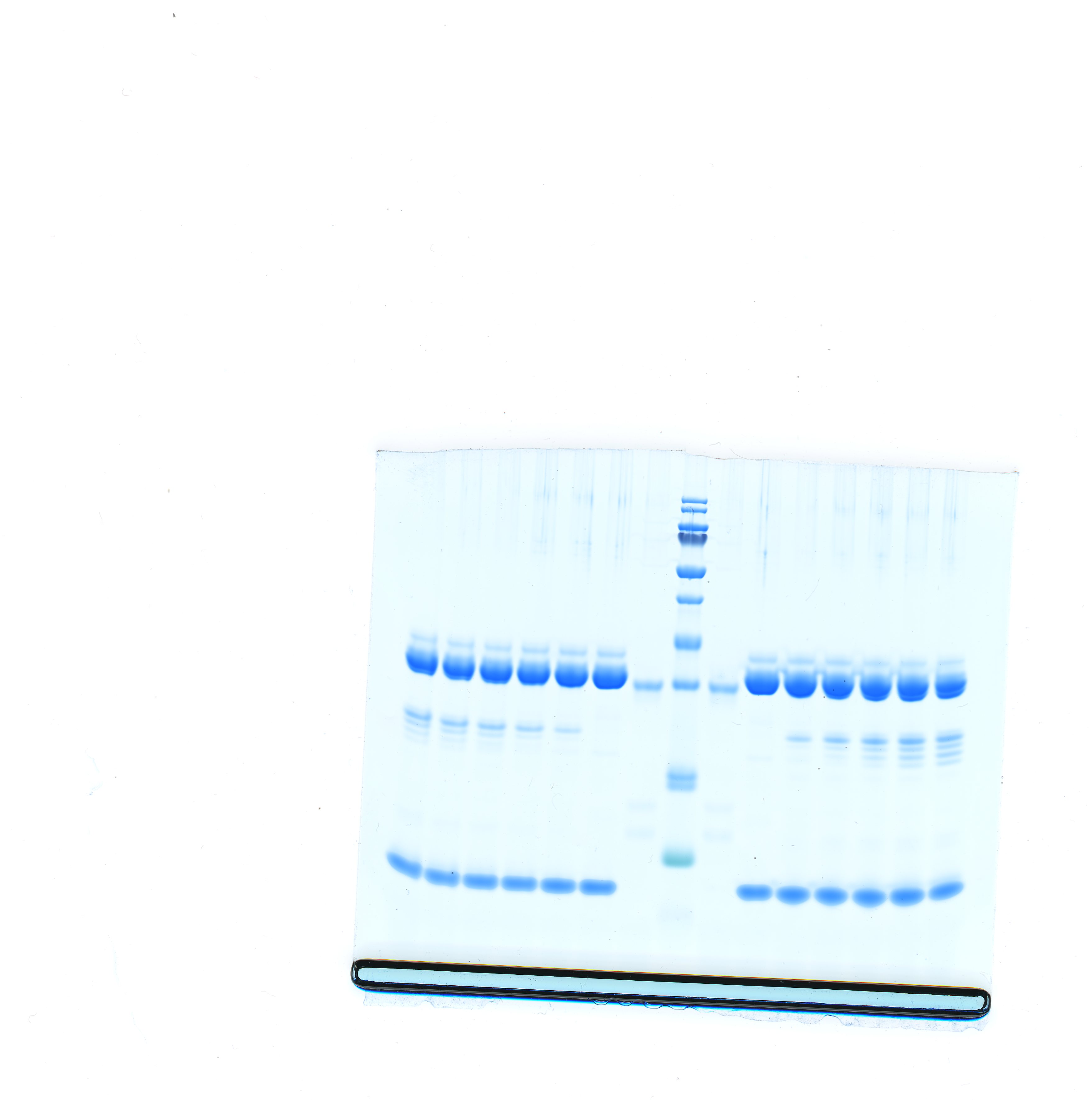

Supplement: Figure 4—figure supplement 1—source data 1. [file elife-83868-fig4-figsupp1-data1.zip › Figure 4-figure supplement 1-CHP3-CBDHis-Ca&Mg-uncropped.jpg]

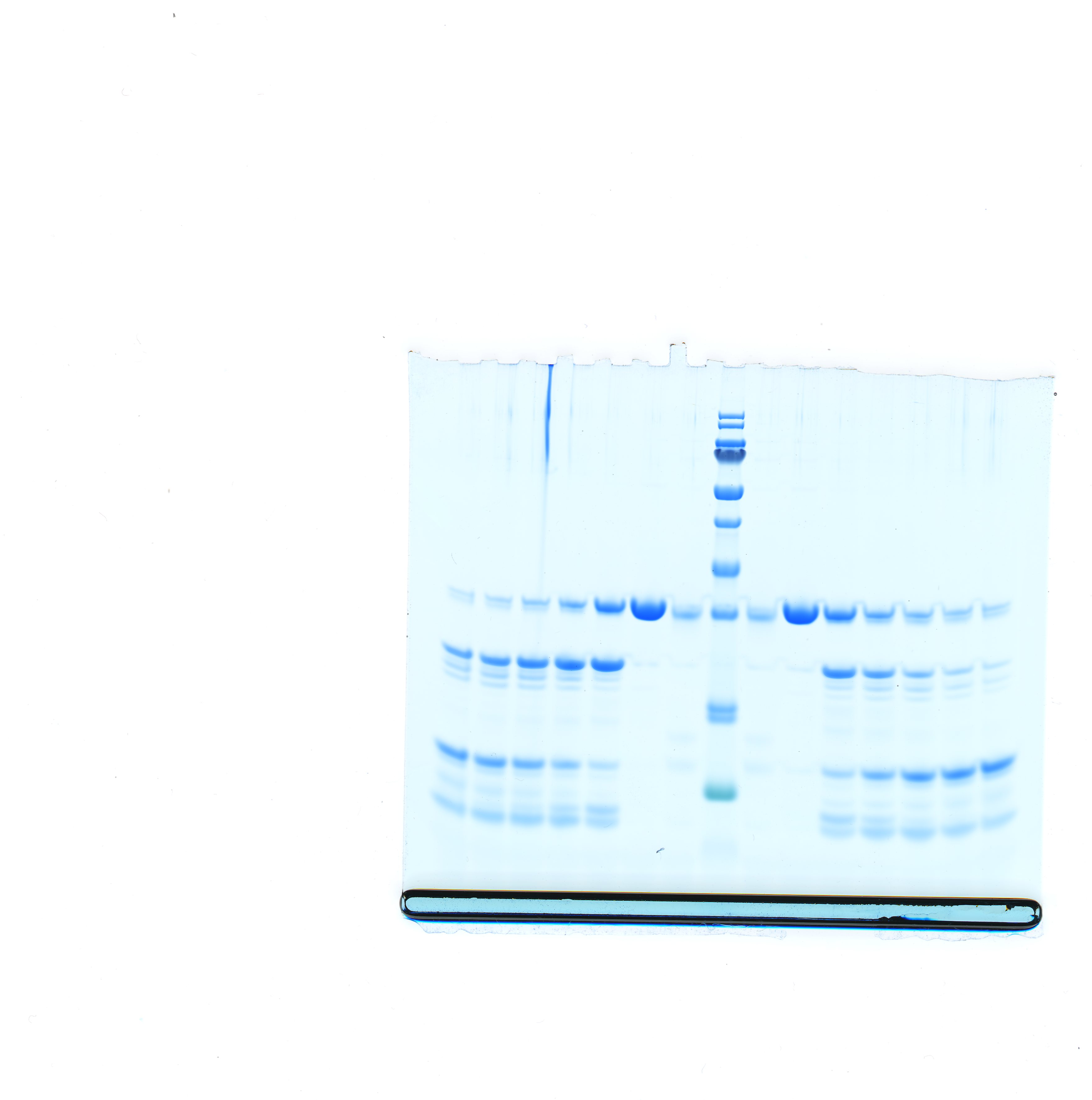

Supplement: Figure 4—figure supplement 1—source data 1. [file elife-83868-fig4-figsupp1-data1.zip › Figure 4-figure supplement 1-CHP3myr-Ca&Mg-uncropped.jpg]

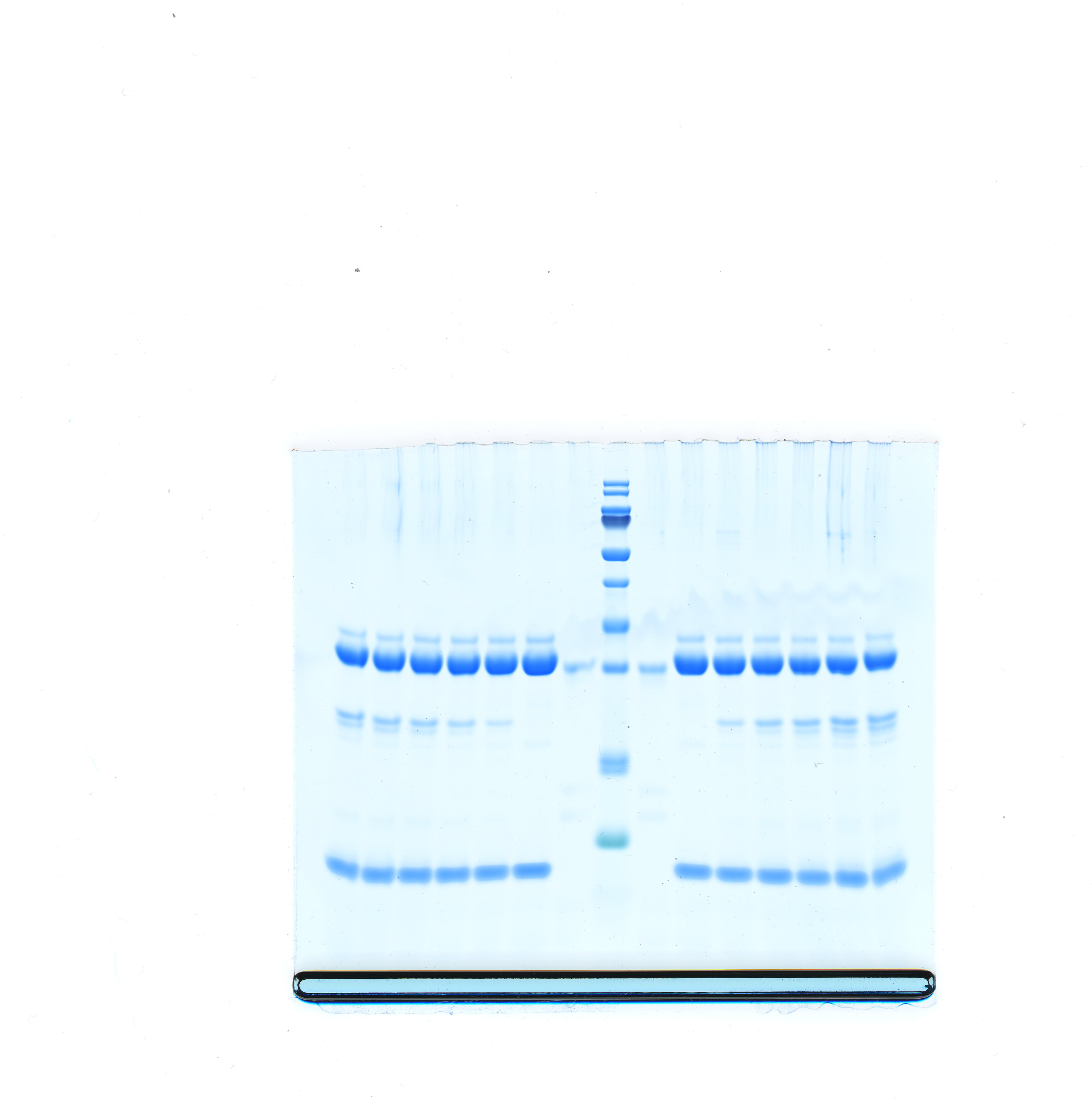

Supplement: Figure 4—figure supplement 1—source data 1. [file elife-83868-fig4-figsupp1-data1.zip › Figure 4-figure supplement 1-CHP3myr-CBDHis-Ca&Mg-uncropped.jpg]

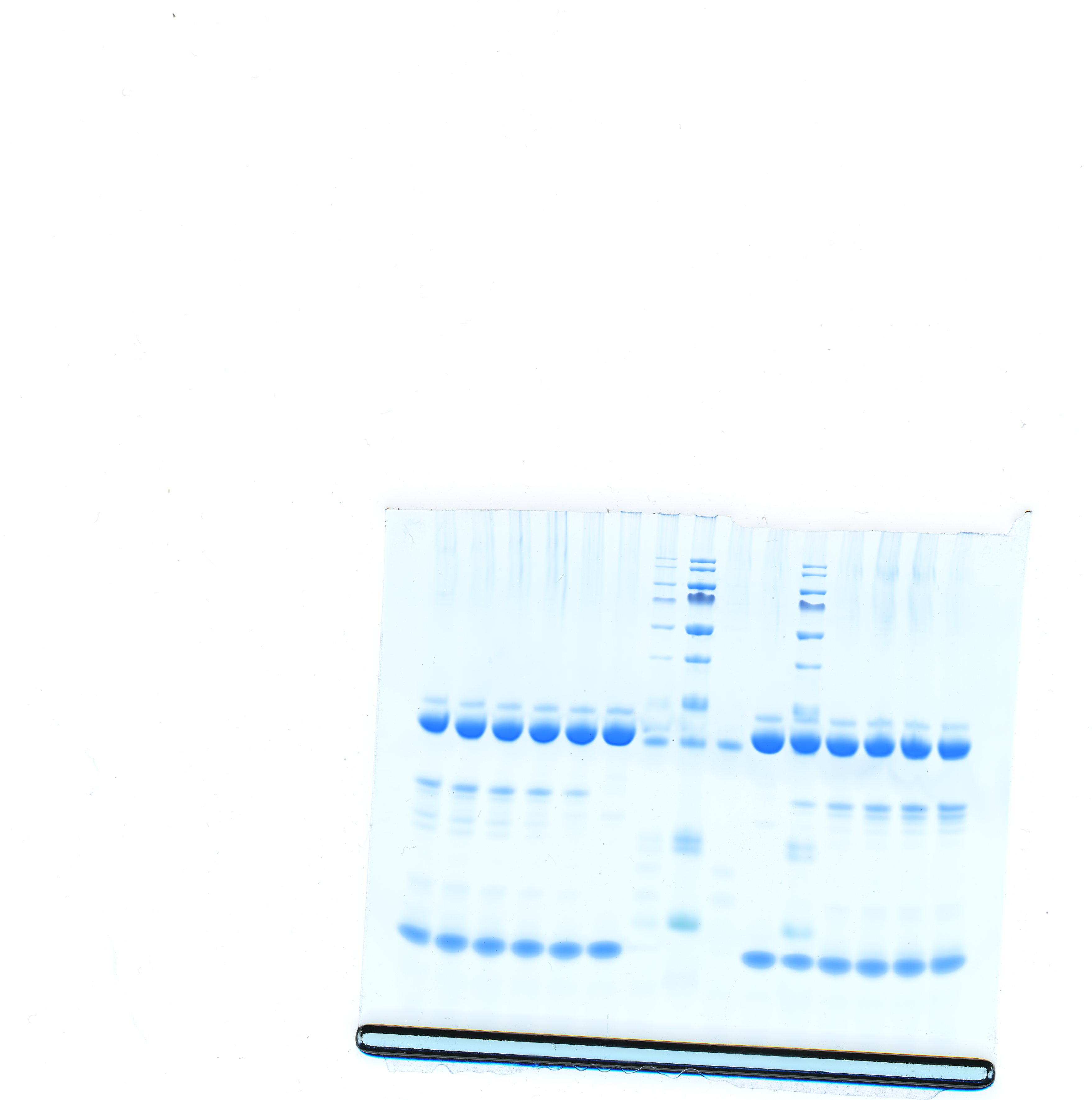

Supplement: Figure 4—figure supplement 1—source data 1. [file elife-83868-fig4-figsupp1-data1.zip › Figure 4-figure supplement 1-CHP3myr-CBDHis-EDTA&CaMg-uncropped.jpg]

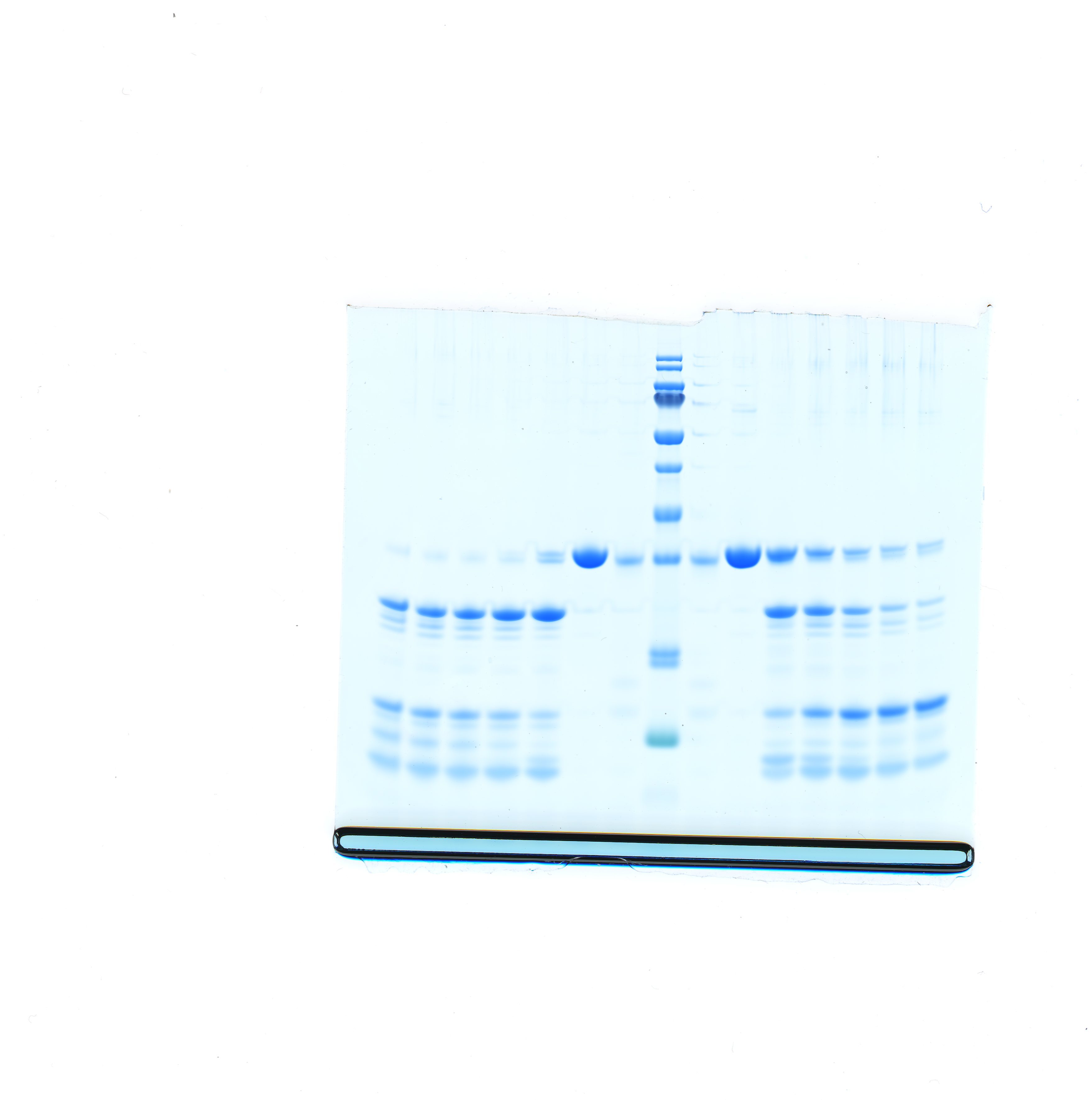

Supplement: Figure 4—figure supplement 1—source data 1. [file elife-83868-fig4-figsupp1-data1.zip › Figure 4-figure supplement 1-CHP3myr-EDTA&CaMg-uncropped.jpg]

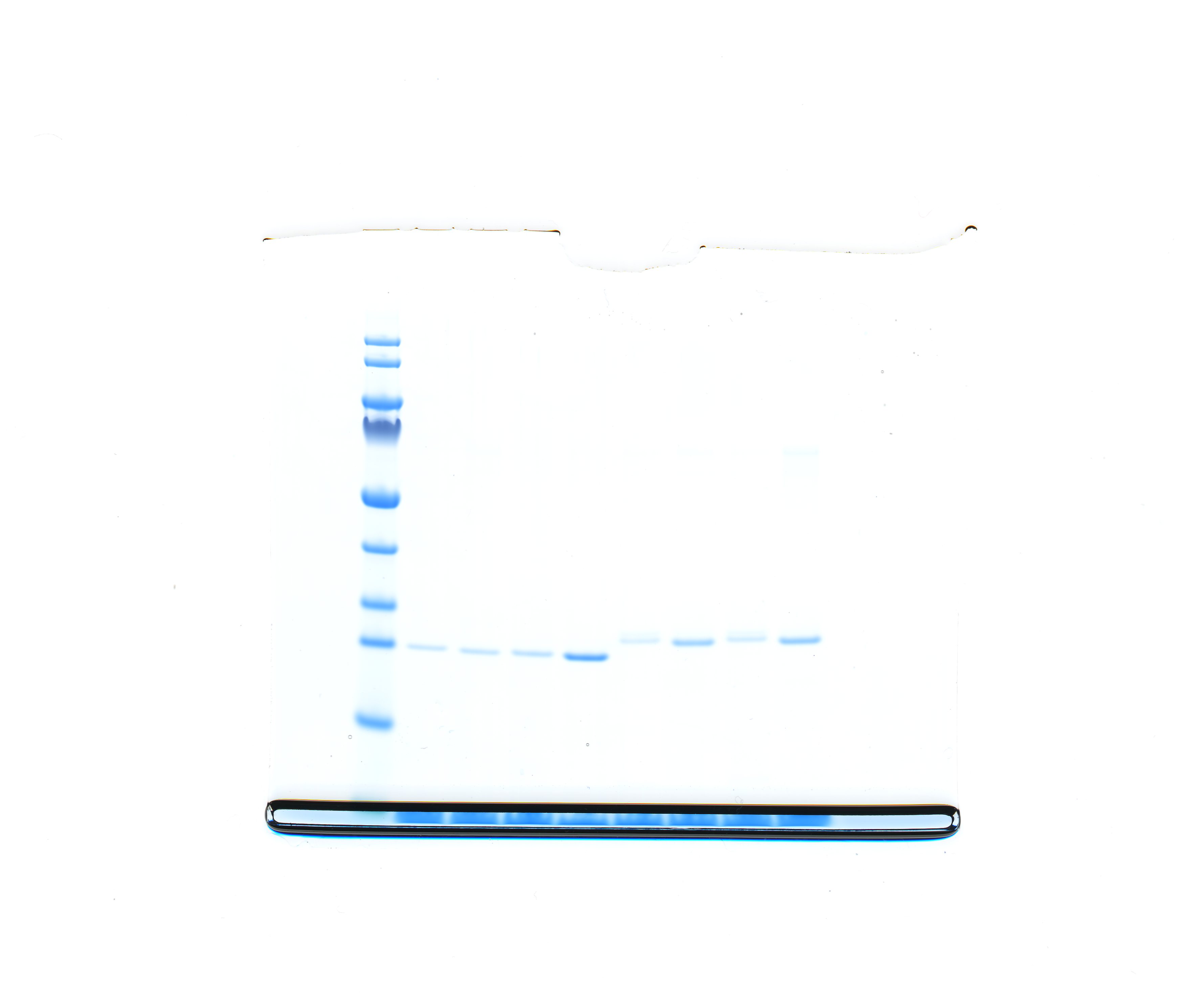

Supplement: Figure 6—source data 1. [file elife-83868-fig6-data1.zip › CHP3-recoverin_replicate2_uncropped.jpg]

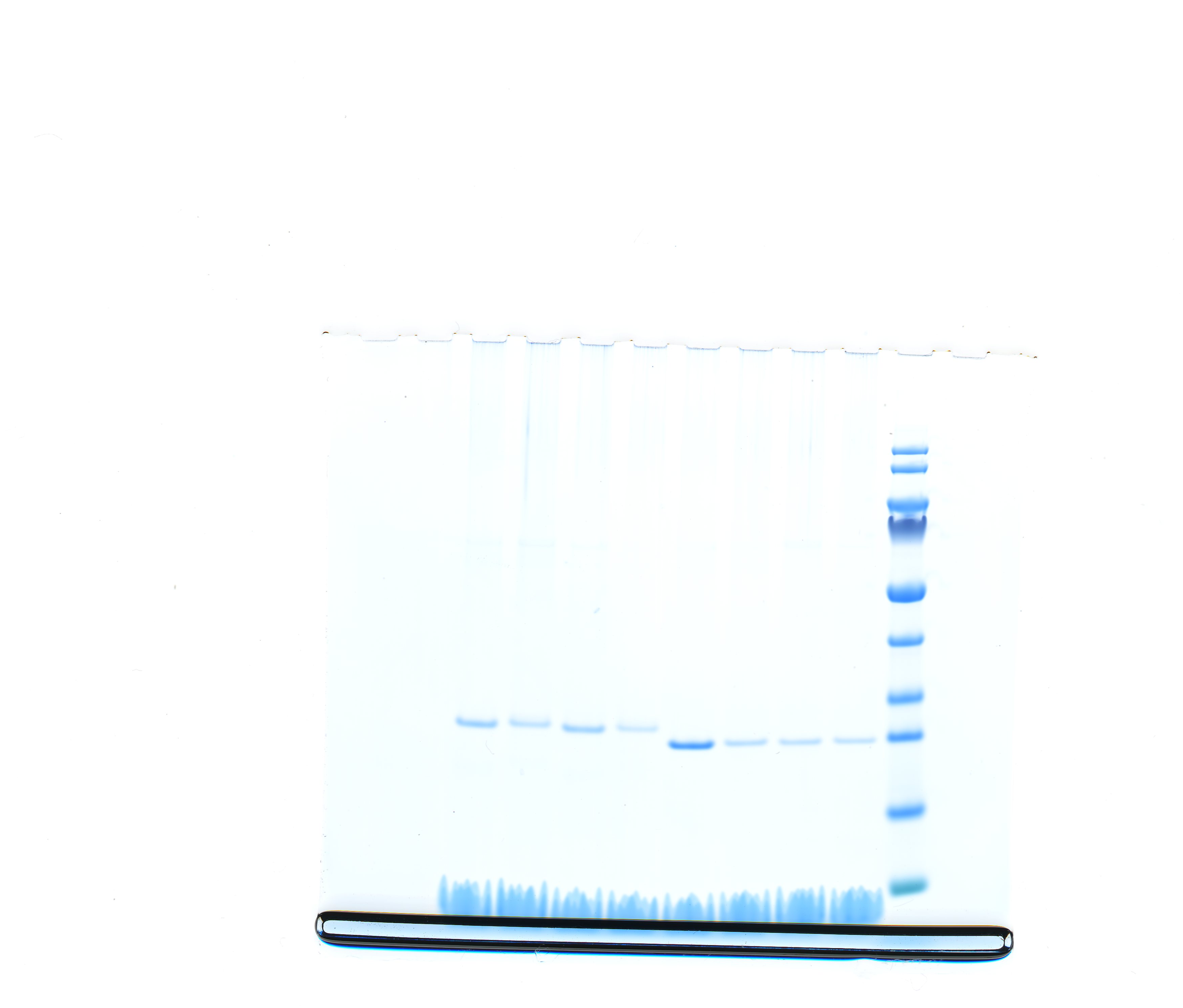

Supplement: Figure 6—source data 1. [file elife-83868-fig6-data1.zip › CHP3-recoverin_replicate3_uncropped.jpg]

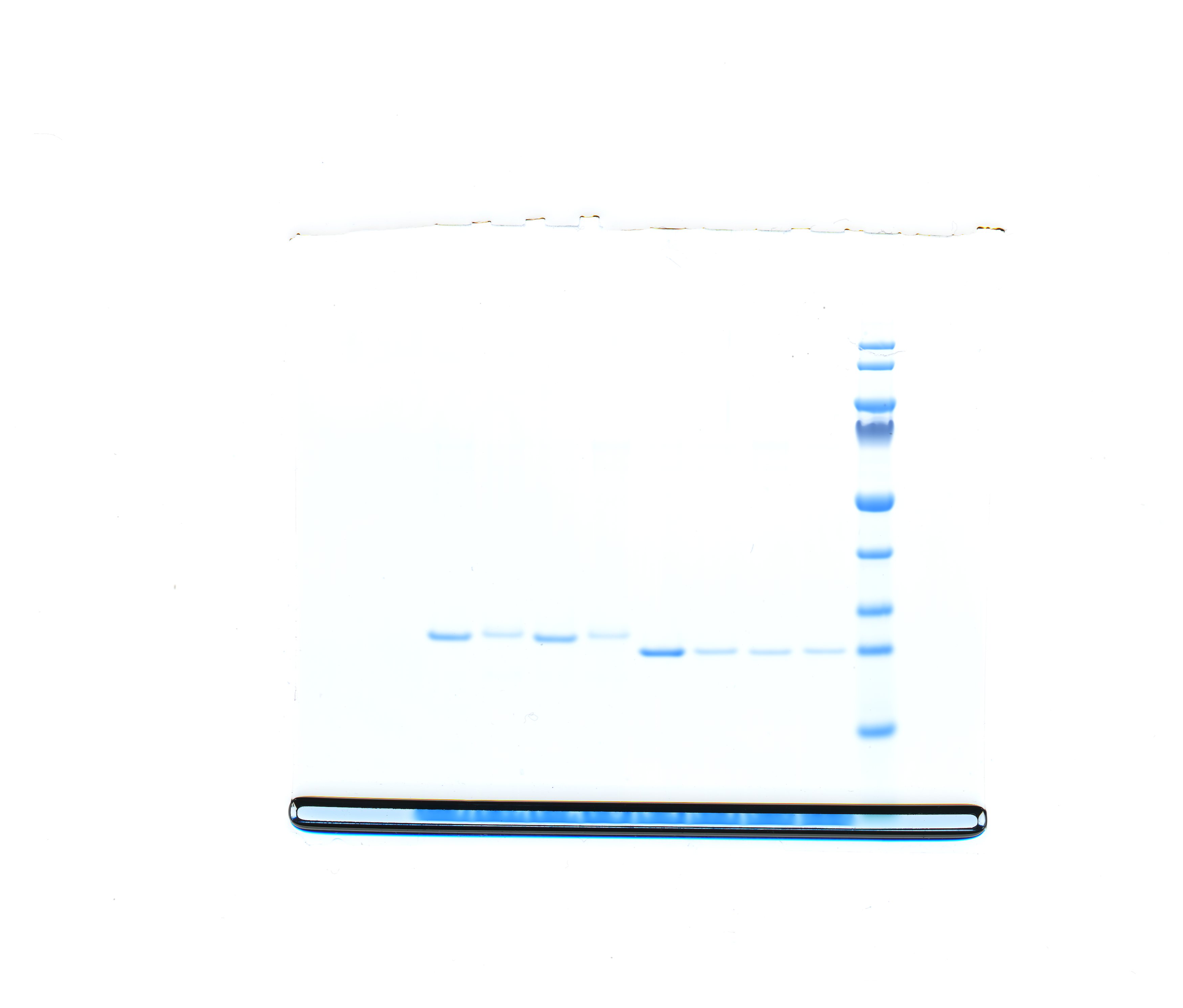

Supplement: Figure 6—source data 1. [file elife-83868-fig6-data1.zip › Figure6A_CHP3-recoverin_replicate1_uncropped.jpg]

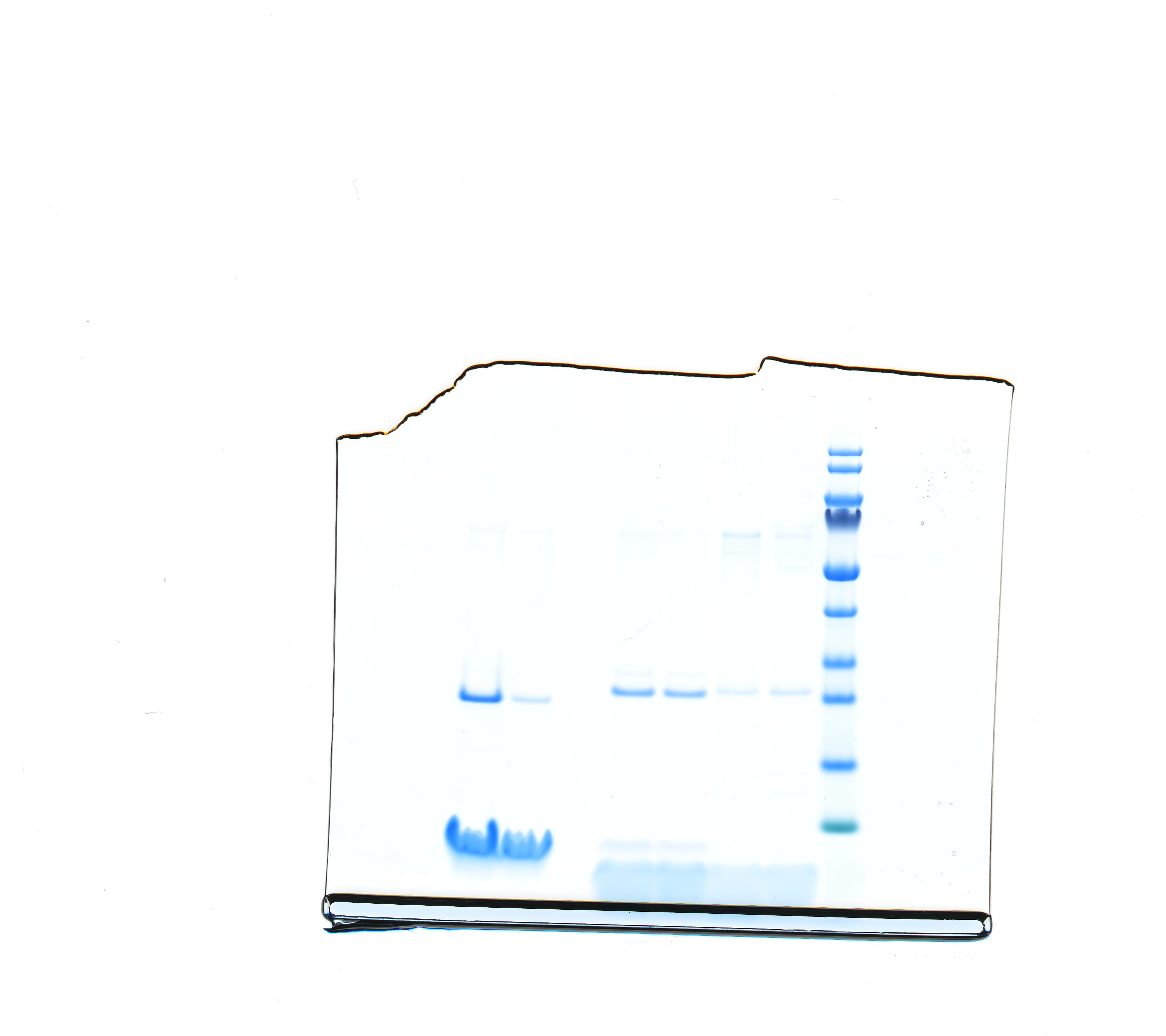

Supplement: Figure 6—source data 1. [file elife-83868-fig6-data1.zip › CHP3CBD_replicate3_uncropped.jpg]

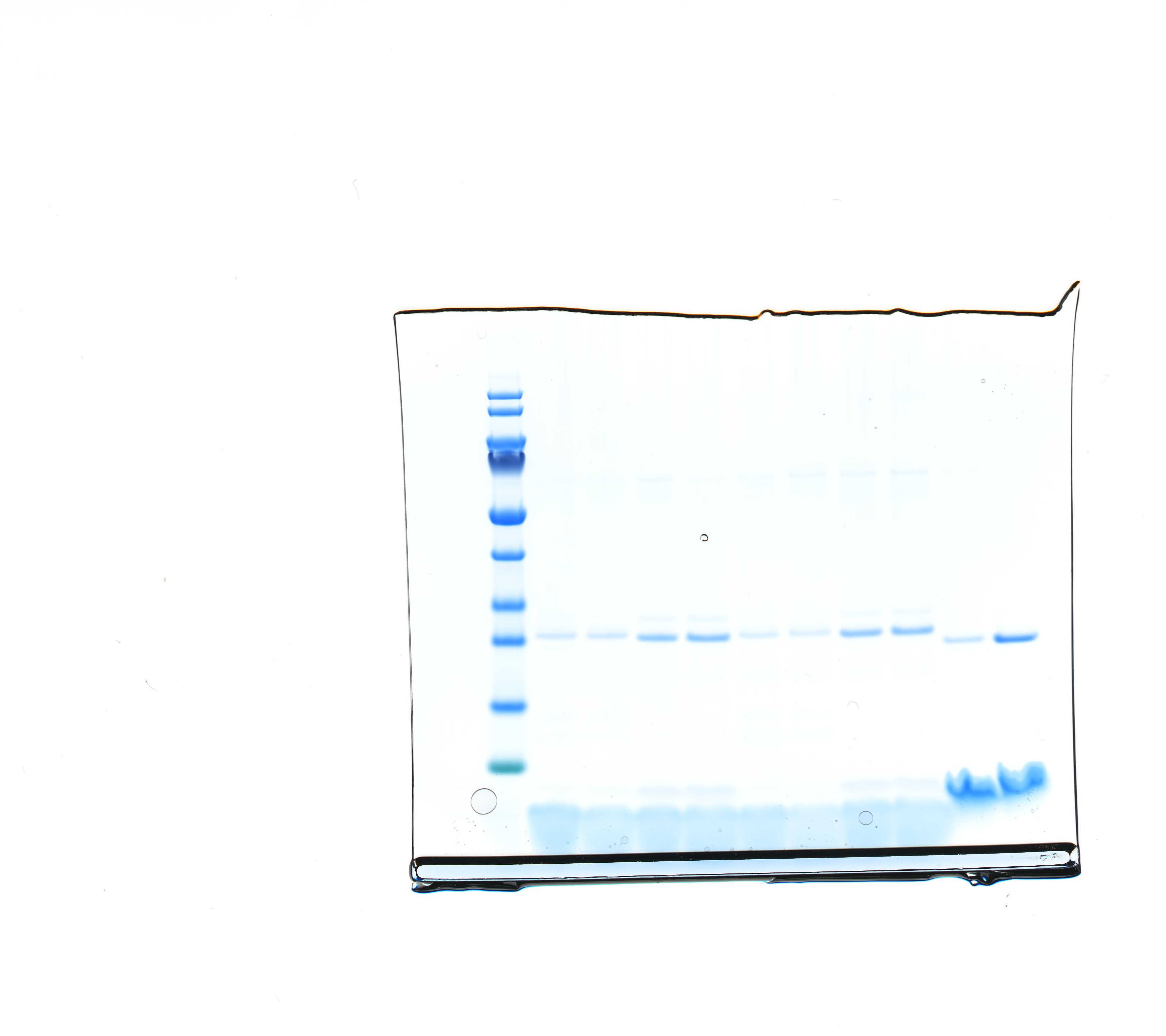

Supplement: Figure 6—source data 1. [file elife-83868-fig6-data1.zip › Figure6A_CHP3CBD_replicate1-2_uncropped.jpg]
