# Supplementary material for: Conformational regulation and target-myristoyl switch of calcineurin B homologous protein 3
Source: eLife. 2023 Jul 12;12:e83868. doi: 10.7554/eLife.83868 (PMC10368425; doi:10.7554/eLife.83868)
Supplement: Figure 4—figure supplement 1—source data 1. [file elife-83868-fig4-figsupp1-data1.zip › Figure 4-figure supplement 1_source_data_1_labeled.pdf]

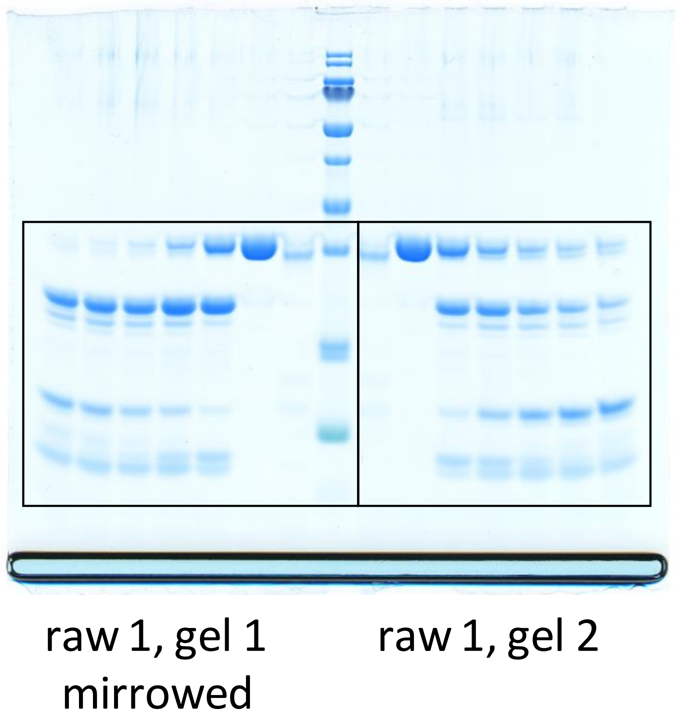
raw 1, gel 1
mirrowed
raw 1, gel 2

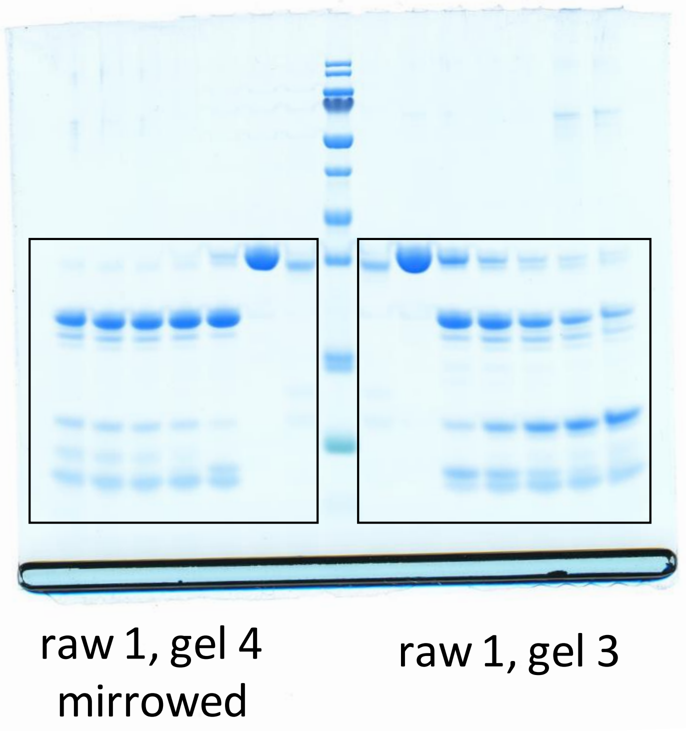
raw 1, gel 4
mirrowed
raw 1, gel 3

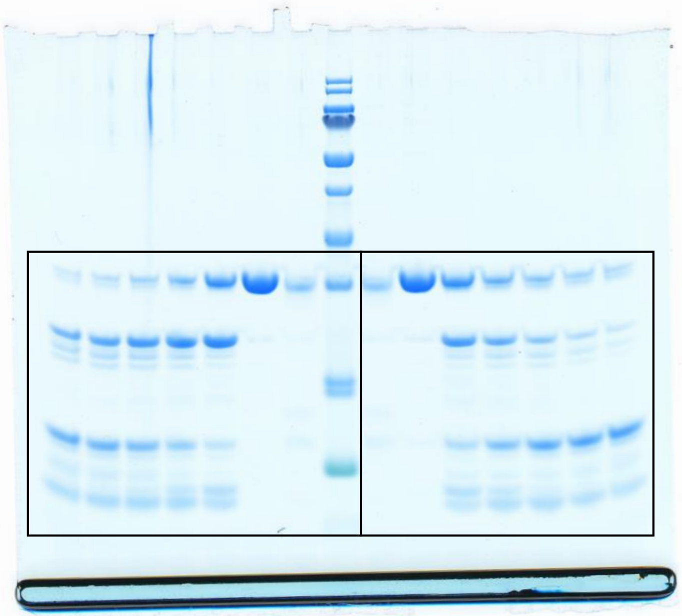

raw 2, gel 1 mirrowed

raw 2, gel 2

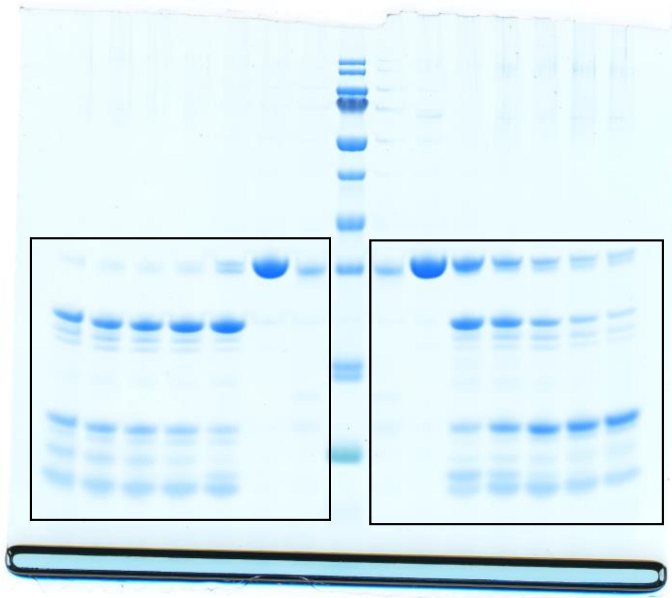

raw 2, gel 4 mirrowed

raw 2, gel 3

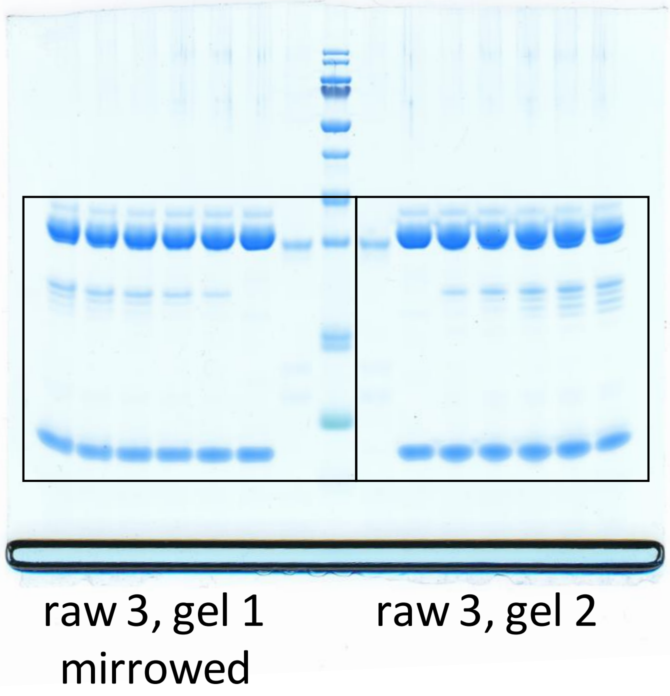
raw 3, gel 1
mirrored
raw 3, gel 2

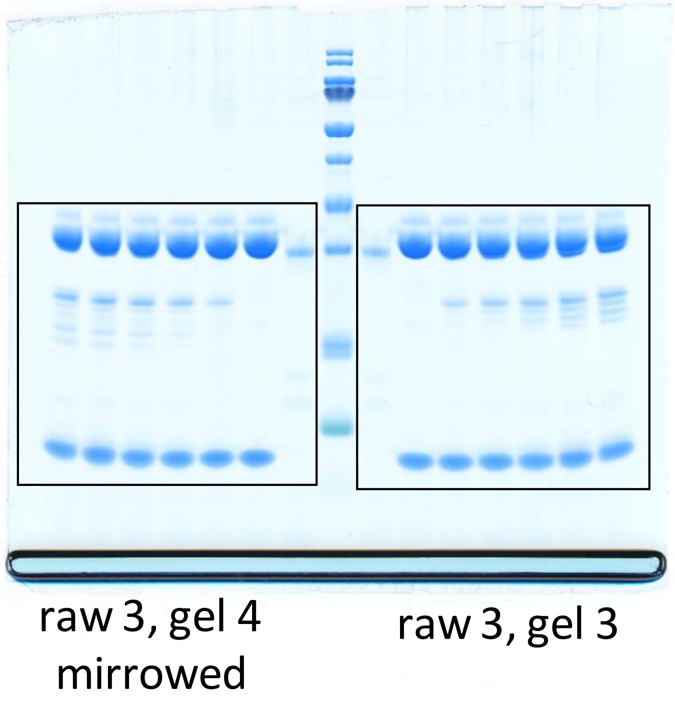
raw 3, gel 4
mirrored
raw 3, gel 3

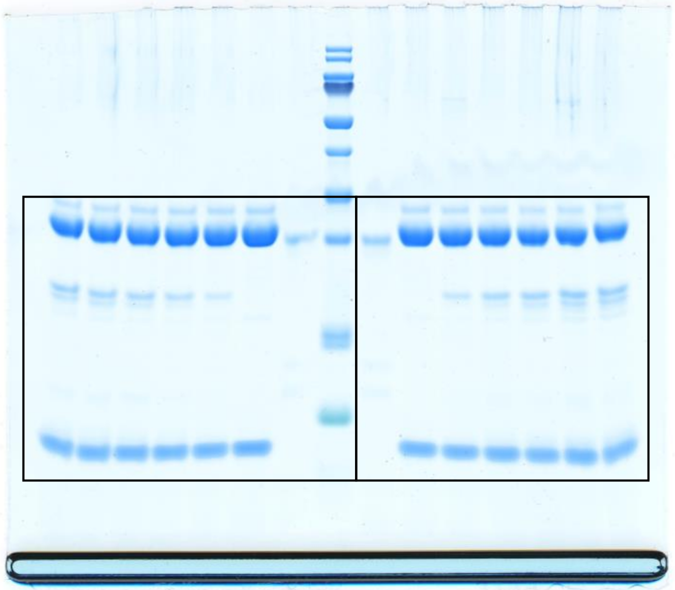
raw 4, gel 1
mirrored
raw 4, gel 2

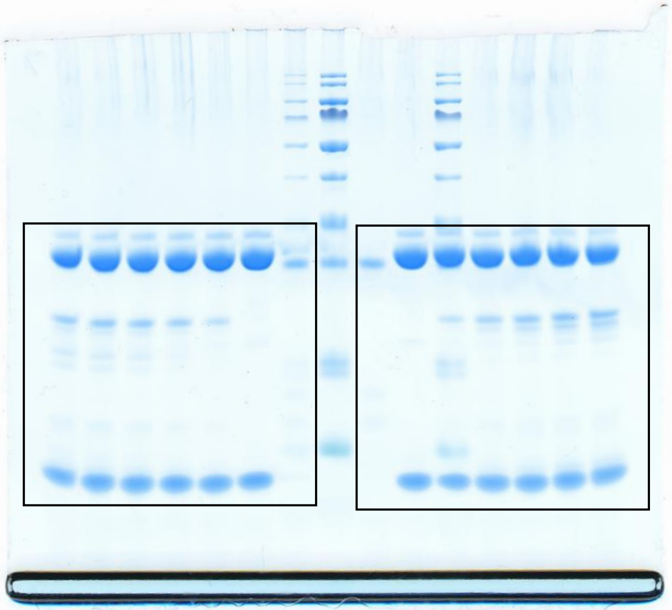
raw 4, gel 4
mirrored
raw 4, gel 3
