# Supplementary material for: Conformational regulation and target-myristoyl switch of calcineurin B homologous protein 3
Source: eLife. 2023 Jul 12;12:e83868. doi: 10.7554/eLife.83868 (PMC10368425; doi:10.7554/eLife.83868)
Supplement: Figure 6—source data 1. [file elife-83868-fig6-data1.zip › Figure6A_labeled.pdf]

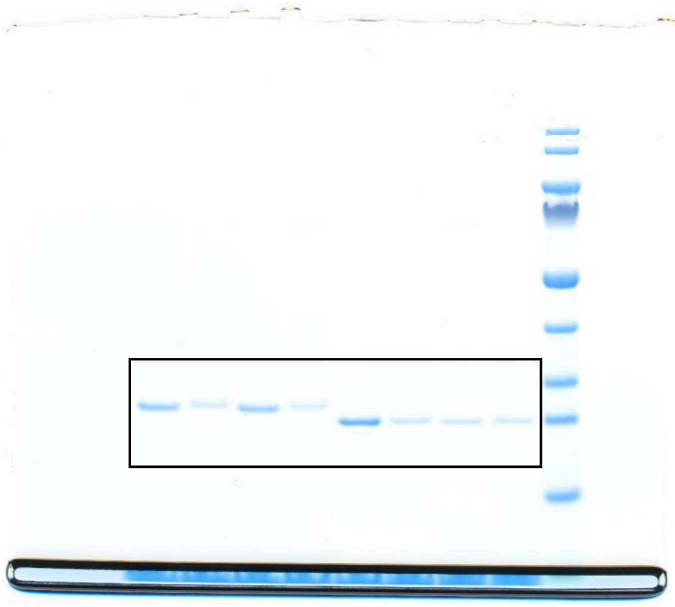

Fig. 6A top
mirrored

Recoverin, CHP3
replicate 1

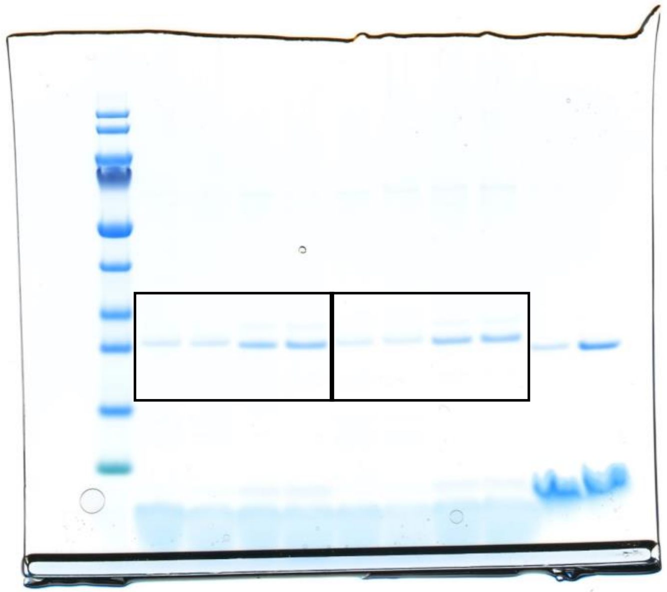

Fig. 6A bottom

CHP3:CBD
replicate 1

CHP3:CBD
replicate 2

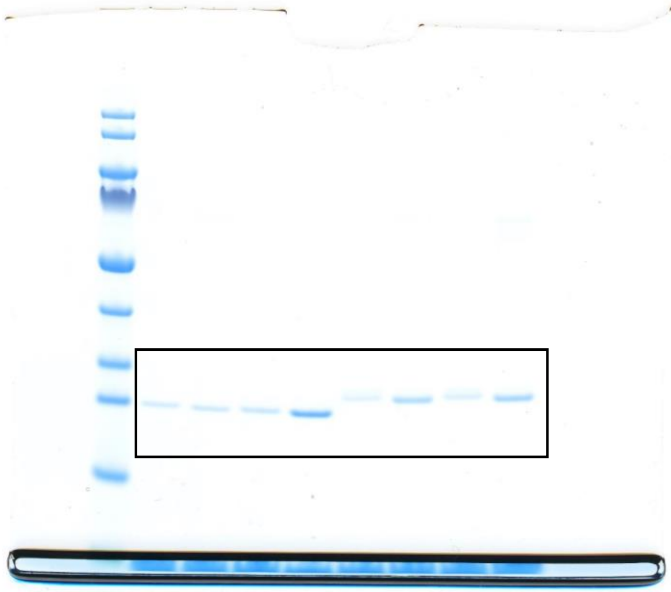

Recoverin, CHP3
replicate 2

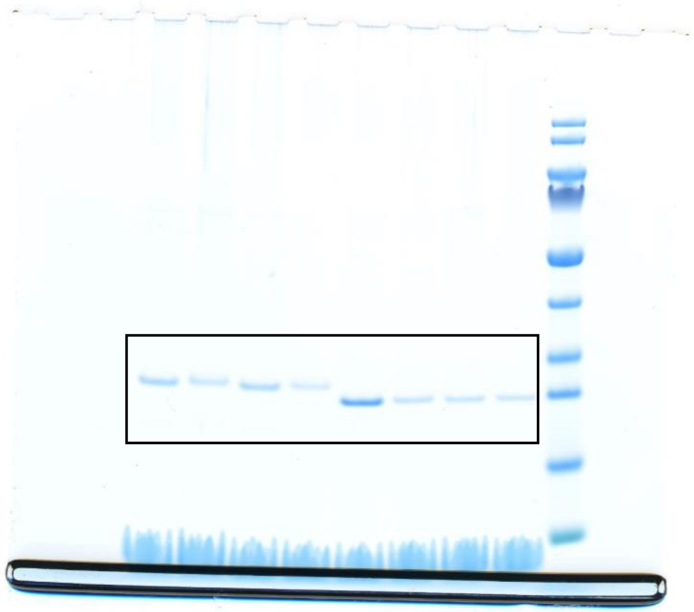

Recoverin, CHP3
replicate 3
mirrored

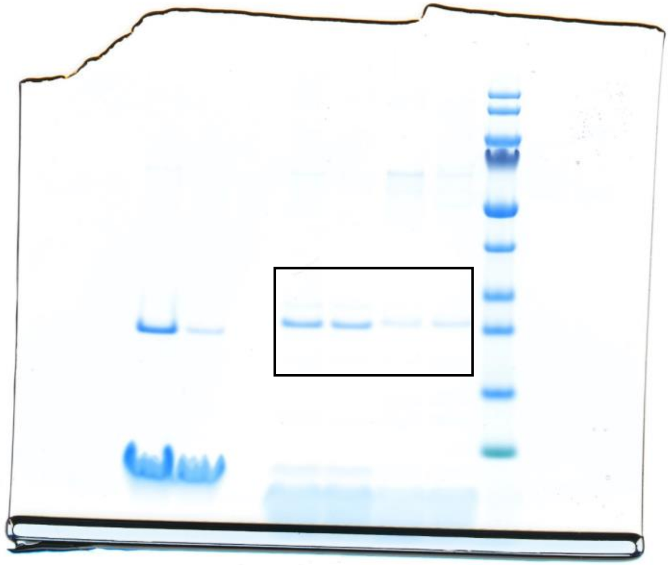

CHP3:CBD
replicate 3
mirrored
